# Printed Potentiometric Nitrate Sensors for Use in Soil

**DOI:** 10.3390/s22114095

**Published:** 2022-05-28

**Authors:** Carol L. Baumbauer, Payton J. Goodrich, Margaret E. Payne, Tyler Anthony, Claire Beckstoffer, Anju Toor, Whendee Silver, Ana Claudia Arias

**Affiliations:** 1 Department of Electrical Engineering and Computer Sciences, University of California, Berkeley, CA 94720, USA; carol_baumbauer@berkeley.edu (C.L.B.); paynme0@berkeley.edu (M.E.P.); atoor@berkeley.edu (A.T.); 2 Department of Mechanical Engineering, University of California, Berkeley, CA 94720, USA; paytongoodrich@berkeley.edu; 3 Department of Environmental Sciences, Policy, and Management, University of California, Berkeley, CA 94720, USA; t.anthony@berkeley.edu (T.A.); cbeckstoffer@berkeley.edu (C.B.); wsilver@berkeley.edu (W.S.)

**Keywords:** nitrate sensors, potentiometric sensors, precision agriculture, printed sensors, agricultural sensors, ion-selective membrane, chemical sensors, soil nitrate monitoring

## Abstract

Plant-available nitrogen, often in the form of nitrate, is an essential nutrient for plant growth. However, excessive nitrate in the environment and watershed has harmful impacts on natural ecosystems and consequently human health. A distributed network of nitrate sensors could help to quantify and monitor nitrogen in agriculture and the environment. Here, we have developed fully printed potentiometric nitrate sensors and characterized their sensitivity and selectivity to nitrate. Each sensor comprises an ion-selective electrode and a reference electrode that are functionalized with polymeric membranes. The sensitivity of the printed ion-selective electrodes was characterized by measuring their potential with respect to a commercial silver/silver chloride reference electrode in varying concentrations of nitrate solutions. The sensitivity of the printed reference electrodes to nitrate was minimized with a membrane containing polyvinyl butyral (PVB), sodium chloride, and sodium nitrate. Selectivity studies with sulphate, chloride, phosphate, nitrite, ammonium, calcium, potassium, and magnesium showed that high concentrations of calcium can influence sensor behavior. The printed ion-selective and reference electrodes were combined to form a fully printed sensor with sensitivity of −48.0 ± 3.3 mV/dec between 0.62 and 6200 ppm nitrate in solution and −47 ± 4.1 mV/dec in peat soil.

## 1. Introduction

Nitrate (${\mathrm{NO}}_{3}^{-}$) is both a critical nutrient for plant growth and a potentially harmful pollutant of drinking water, yet tools for monitoring nitrate broadly over time and space are inadequate. In agriculture, nitrogen—often in the form of nitrate (NO_3_-N)—is a key component of fertilizer. Grain growers apply up to a few hundred pounds of nitrogen per acre, depending on the crop and field conditions [1]. At a cost of tens of cents to a dollar (USD) per pound, with prices rapidly increasing in recent months, it is the second highest cost for many crops, surpassed only by seeds [2]. Nitrate fertilizer is conventionally applied uniformly across a field, despite studies that have shown that the existing nitrate concentration in soil can vary significantly on the order of tens of meters. Precision agriculture practitioners aim to designate site-specific management zones to direct more efficient nitrogen application, but measurement tools are limited. Optical remote sensing can be used to estimate nitrogen in growing plant material, but to obtain measurements of NO_3_-N in soil, a soil sample must be collected and taken back to a laboratory, for analysis via chromatography or spectrographic methods [3,4]. Such measurements are highly accurate, but they are also expensive, labor-intensive, and give data for only one point in time and space. Nitrate is highly mobile, so concentrations change over time. Models can be developed to estimate nitrate fluxes based on measurements at the beginning and end of a season, but these rely on many estimations and assumptions [5].

The excessive application of nitrate also has harmful environmental consequences. Nitrate easily leaches into groundwater, where it contaminates well water used for drinking [6]. Excess nitrate (above 10 ppm) in drinking water is known to cause adverse outcomes to human health [7,8]. Nitrates can also run off and accumulate in surface bodies of water, which can lead to harmful algal blooms and eutrophication [9].

**Figure 1:**
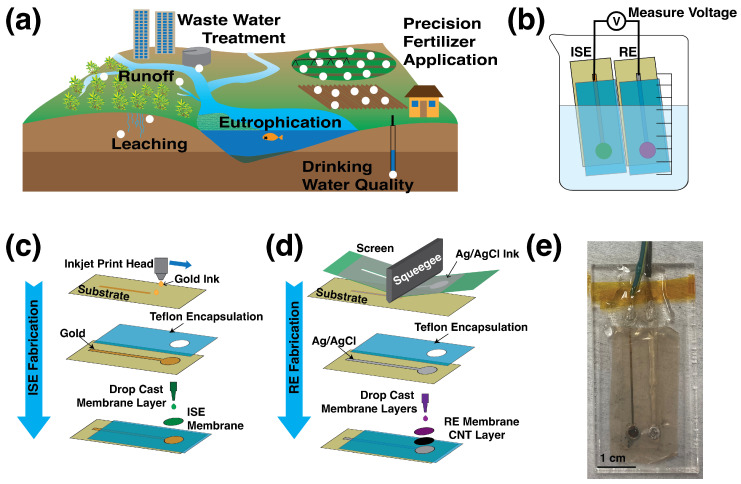
(**a**) Printed sensors could be widely deployed to map nitrate from fertilizer application to runoff. (**b**) The potential difference between the printed ion-selective and printed reference electrodes is determined by the nitrate concentration in the solution, and this potential is read as the sensor’s output. (**c**) Ion-selective electrodes are made by inkjet printing gold onto a substrate, and encapsulating the trace with a teflon tape. The membrane solution is drop-cast onto the exposed area of the electrode. (**d**) Reference electrodes are made by screen printing Ag/AgCl ink onto the substrate, and the trace is encapsulated with teflon. The carbon nanotube transducing layer is drop-cast first, followed by the PVB/salt membrane. (**e**) A fully printed sensor mounted on acrylic backing and with wires attached is ready for use in soil tests.

To prevent the consequences of excess nitrate in the environment, nitrate levels must be monitored. To better quantify the nitrate problem, and better tailor nitrogen fertilizer inputs in agriculture, more frequent measurements at high spatial resolution over large areas are needed. This could be achieved with a network of distributed sensors, as illustrated in Figure 1a, where each white circle represents a nitrate sensor that could provide real-time concentration data at locations where nitrate enters and accumulates in the ecosystem.

Environmental quality monitoring and precision agriculture require nitrate sensors that are mass-producible, easy-to-read, involve few or no moving parts, and are robust enough to survive field deployment and soil insertion. Printed solid-state potentiometric ion-selective electrodes have the potential to meet these criteria. The use of printing methods for the sensor fabrication offers several advantages, such as low cost, high throughput, and ease of fabrication. Potentiometric sensors are composed of two electrodes: an ion-selective electrode (ISE) and a reference electrode (RE). The signal output is the potential difference between the two electrodes at zero-current conditions, as shown in Figure 1b. An ISE has a polymer membrane doped with an ionophore—a chemical designed to selectively and reversibly bind to the ion of interest [10,11,12,13,14,15,16,17,18]. As the concentration of the ion of interest, ${\mathrm{NO}}_{3}^{-}$ in this case, in the sample solution increases, the potential that develops at the boundary between the ion-selective membrane and the sample increases. Buhlmann and Chen provide a clear background explanation of the chemistry and thermodynamics governing this process [19]. The result is that the potential at the ISE is described by the Nernst equation:

(1) $$E=E_{0}+2.3026\frac{RT}{zF}log_{10}\left(a_{ion}\right)$$

where *E* is the potential measured across the electrodes, $E_{0}$ is the cell potential, *R* is the ideal gas constant, *T* is the temperature, *F* is Faraday’s constant, *z* is the charge of the ion of interest, and $a_{ion}$ is the ion activity. The ion activity is a function of the concentration of the ion in solution and the activity coefficient, which is 1 for sufficiently dilute solutions. Thus, an ideal potentiometric sensor for a monovalent anion, such as ${\mathrm{NO}}_{3}^{-}$, at room temperature is expected to exhibit a −59.1 mV change for every factor of ten increase in ${\mathrm{NO}}_{3}^{-}$ concentration. The potential difference measured between the ISE and RE is the sum of the potentials developed at each metal–metal, metal–solid, and solid–liquid boundary. Ideally, only the ion-selective membrane–sample boundary potential depends on the nitrate concentration; the other boundary potentials are accounted for by $E_{0}$ in the Nernst equation.

REs are typically made of silver/silver-chloride (Ag/AgCl) and maintain a constant potential in varying ionic environments [20]. Commercially available reference electrodes are glass tubes with a silver-chloride-coated silver wire in the center, surrounded by a saturated solution of potassium chloride (KCl). A porous ceramic membrane keeps the filling solution inside the tube. Most potentiometric sensor studies rely on such liquid-based reference electrodes. Solid-state reference electrodes are more suitable for field deployment because they have the neither the liquid filling nor the fragile glass tube, but solid-state references are typically less stable than commercial references. Significant challenges remain in the development of solid-state reference electrodes, yet relatively few studies characterize printed references with the same rigor as ISEs [21].

Because soil is a complex environment containing many ions that could interfere with a nitrate sensor, selectivity is particularly important for sensors intended for use in soil [22]. Selectivity studies for anion ISEs such as nitrate typically focus on ions from the Hoffmeister series, which are most likely to interfere. While these characterizations are important, soil can contain high concentrations of other ions, so both the ISE and RE must be characterized for sensitivity to ions found in soil. ISEs obtain their selectivity from specially designed synthetic ionophores, although even these have nonidealities. Ions present in the environment can also interfere with ion binding sites or charge transport materials in the membrane, hindering the functionality of the ISE.

Table 1 compares potentiometric nitrate-selective electrode measurements. Several works use scalable fabrication techniques such as screen or stencil printing to fabricate electrodes and drop-cast membranes similar to this work. Three of these use commercial references and demonstrate measurements in liquid samples. Three more use solid-state references and show applications in soil or soil slurries; however, only one work characterizes the reference electrode’s stability, and none report stability in varying nitrate concentrations or the impact of interfering ions. Another group of works explore transducing layer materials for improved stability. These works are based on glassy carbon electrodes, do not use printing techniques, and most do not demonstrate performance in real-world conditions.

In this work, we characterize the intermediate steps between nitrate ISE demonstration in aqueous solution and the deployment of a fully printed sensor in the field. The sensitivity of the ISEs was measured against commercially available REs and demonstrated a near-Nernstian response to nitrate. Printed REs were optimized for stability across a range of nitrate concentrations. Both ISEs and REs were independently tested in solutions containing eight potentially interfering ions, which were chosen for their prevalence in soil. Calcium had the most significant impact on both ISEs and REs. The printed nitrate ISEs were paired with printed REs to create fully printed nitrate sensors, which are slightly less sensitive to nitrate in aqueous solutions than printed ISEs paired with glass references. Fully printed nitrate sensors were measured in a high-organic-matter field soil, and demonstrated sensitivity equal to their sensitivity in solution.

## 2. Materials and Methods

### 2.1. Ion-Selective Electrode Fabrication

ISEs were fabricated according to the process illustrated in Figure 1c. Gold electrodes, which were 3.5-mm-diameter circles connected to a 1-mm-wide trace, were printed on 25-μm-thick PQA2 PEN using Harima Nanopaste(Au) NPG-J gold ink in a Dimatix DMP-2850 inkjet printer (Fujifilm Dimatix, Santa Clara, CA, USA) using a 10 pL cartridge and no platten heating. Printed gold electrodes were sintered at 250 °C for 50 min and then encapsulated with 75-μm-thick laser-cut Teflon tape with circular windows of 5 mm diameter for the active area. The window in the encapsulant was larger than the electrode to allow space for the membrane to seal to the substrate, preventing bubbles or delamination of the membrane. ISE membranes were fabricated by mixing 5.2 wt% nitrate ionophore VI, 47.1 wt% dibutyl phthalate, 0.6 wt% tetaroctylammonium chloride, and 47.1 wt% PVC. A total of 0.2 g of this mixture was dissolved in 1.3 mL of tetrahydrofuran (THF). Sixteen μL of the membrane solution was drop-cast on the printed gold electrode surface. The resulting ISE was dried in a fume hood for 15 min.

### 2.2. Reference Electrode Fabrication

Printed RE fabrication is outlined in Figure 1d. Ag/AgCl electrodes with the same geometry as the gold electrodes were screen-printed on 100-μm-thick PET using Engineered Materials Systems, Inc. CI-4001 ink (Delaware, OH, USA). Three layers of ink were printed; each layer was dried before the next was printed. Printed Ag/AgCl electrodes were then annealed at 120 °C in a vacuum oven for 2 hours and encapsulated with laser-cut Teflon tape 75 μm thick.

The REs employed a CNT transducer between the Ag/AgCl electrode and the membrane. This transducer was composed of 0.01 g of CNT (iP-Single-Walled Carbon Nanotubes from Carbon Solutions, Inc., Riverside, CA, USA) and 0.05 g of F127 (poly(ethylene glycol)-block-poly(propylene glycol)-block-poly(ethylene glycol) diacrylate) dissolved in 10 mL of THF, which were sonified for 1 hour in an ice bath using a Branson Digital Sonifier probe. The resulting mixture was deposited on the printed REs’ surface as 4 μL total in two separate 2 μL increments. The RE membrane was made by dissolving 1.58 g of Butvar B-98 (poly(vinyl butyral) (PVB), 1.00 g of NaCl, and 1.00 g of NaNO_3_ in 20 mL of methanol. This mixture was sonified for 30 min in an ice bath. The resulting solution was deposited on top of the CNT transducer as 6 μL total in three separate 2 μL increments. Unless otherwise noted, all chemicals used in both ISE and RE membranes were obtained from Millipore Sigma (St. Louis, MO, USA).

Fully printed sensors used in soil studies were attached to an acrylic block for mechanical stability. Moreover, 8331D silver conductive epoxy (MG Chemicals, Burlington, ON, Canada) was used to connect wires, and the joint was encapsulated by Gorilla epoxy. Figure 1e shows a photograph of the printed sensor used in soil tests.

### 2.3. Measurements in Solution

Commercial Ag/AgCl electrodes with liquid filling solution were obtained from Millipore Sigma (Z113107). To perform sensitivity measurements, ${\mathrm{NaNO}}_{3}$ was dissolved in deionized water, and diluted to 0.05, 0.1, 0.2, 0.5, 1, 2, 5, 10, 20, 50, and 100 mM concentrations. Solutions for selectivity experiments were made with powdered Na_2_SO_4_, NaNO_2_, KCl, MgCl_2_, Ca(NO_3_)_2_, NH_4_Cl, Na_3_PO_4_, and NaCl obtained from Millipore Sigma. Prior to measurement, electrodes were conditioned for at least two hours in 100 mM NaNO_3_. Chronopotentiometery was performed using the Keithley 2400 Series SourceMeter, Keysight B2987A Electrometer/High Resistance Meter, and Ivium-n-Stat from Ivium Technologies B.V. (Eindhoven, The Netherlands).

### 2.4. Soil Measurements

For the measurements in soil, a set of six small pots each containing 50 g of peat soil were prepared. The soil was an agricultural peat soil from Bouldin Island in the Sacramento–San Joaquin Delta, California [35,36]. Each container was watered to 50% soil moisture by mass using pure water, or 1, 10, 100, or 1000 mM nitrate solution. KNO_3_ was used as the source of nitrate. The sensors were connected to a Campbell Scientific CR1000 data logger. The sensors were inserted into each container and their potential recorded every 5 s until the output stabilized for at least 3 min per concentration. After measurement in one container, the sensor was removed, rinsed with deionized water, and inserted into the next container.

A post-sensor test KCl extraction was conducted on each soil sample to determine the total extractable nitrate concentration in each soil treatment. Approximately 15 g of each soil sample was added to 75 mL of 2M KCl solution and shaken for 1 hour at 180 rpm. Samples were subsequently filtered through pre-washed Whatman 1 filter paper (Cytiva, Marlborough, MA, USA) and extracts were frozen until colorimetric ${\mathrm{NO}}_{3}^{-}$ analysis (EPA-127-A Rev 8) could be performed using a Seal AQ300 Analyzer (Seal Analytical, Mequon, WI, USA).

## 3. Results and Discussion

### 3.1. Nitrate-Selective Electrode Sensitivity

**Figure 2:**
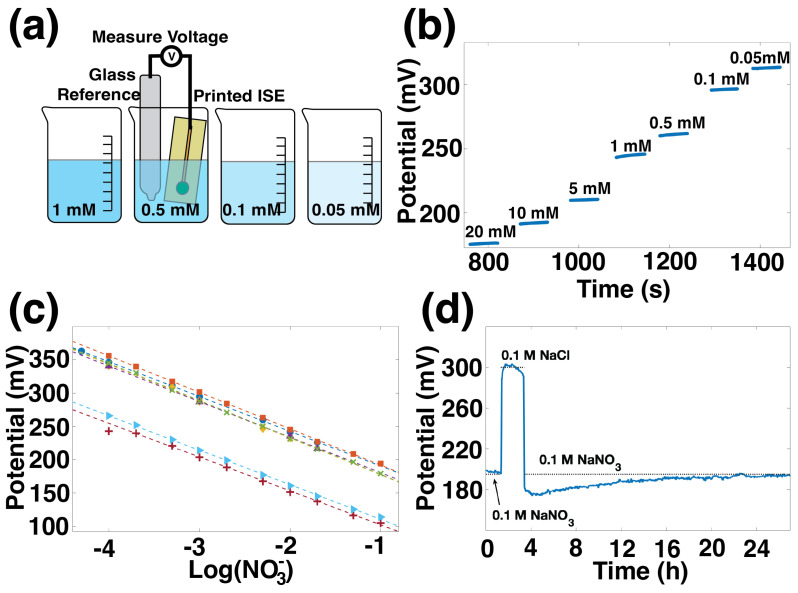
(**a**) Characterization of a printed nitrate-selective electrode against a commercial reference electrode in NaNO_3_ solutions of varying concentrations. (**b**) Potential over time response of a printed nitrate-selective electrode in changing concentrations of nitrate. (**c**) Sensitivity plot of 7 nitrate-selective electrodes overlaid, showing good repeatability and near-Nernstian response of −54.1 ± 2.1 mV/dec. (**d**) Water layer test showing the stability of the nitrate-selective electrode.

The sensitivity of printed ISEs was measured against commercial glass reference electrodes in aqueous solutions, as illustrated in Figure 2a. Figure 2b shows the potential over time for one ISE measured against a glass commercial reference electrode in nitrate solutions between 20 mM and 0.05 mM. This ISE reported a stable potential value less than 30 s after a change in concentration. The data from Figure 2b can alternatively be plotted versus nitrate concentration on a log scale, as shown by the blue circles in Figure 2c. The other lines in Figure 2c represent sensitivity for six additional ISEs in three batches. The average sensitivity for all seven sensors is −54.1 ± 2.1 mV/dec.

The linear region for these sensors is between 0.05 mM and 100 mM. This range is equivalent to 3.1 to 6200 ppm ${\mathrm{NO}}_{3}^{-}$ or 0.7 to 1400 ppm nitrogen (${\mathrm{NO}}_{3}^{-}$-N). Concentrations of nitrate in agricultural fertilizer vary widely depending on crop and soil type as well as fertigation technique, but a few 100 ppm would be a high nitrate concentration in fertilizer [37]. In the United States, the Environmental Protection Agency’s drinking water quality standards specify a maximum of 10 ppm ${\mathrm{NO}}_{3}^{-}$, and some studies have shown an increased risk of certain health conditions for water with 5 ppm ${\mathrm{NO}}_{3}^{-}$ or greater. The sensors presented here cover nitrate concentrations from drinking water to concentrated fertilizer.

In Figure 2c, the sensitivity curves for different sensors are offset one from another. This variation in $E_{0}$ is common in ISEs and means that each sensor must be individually calibrated prior to use. $E_{0}$ variation has a variety of causes, many of which are summarized by Hu et al. [38]. Properly, $E_{0}$ is the potential at ion activity of 1, which is outside of the linear range of the sensors. $E_{0}$ values presented here were calculated using the potential at 1 mM ${\mathrm{NO}}_{3}^{-}$ concentration. Within one batch of ISEs, the $E_{0}$ variation was found to be 12.5 mV. The measurements for one batch were done with each ISE paired with one of several different commercial reference electrodes. While nominally identical, the commercial reference electrodes’ potentials are up to 11 mV different from each other. This difference in commercial references’ performance is consistent with $E_{0}$ values obtained within a batch of ISEs. The batch to batch variation is 83 mV over six batches. This significant variation may be due to variation in the membrane drying and sections of crystallized PVC in the membranes, as suggested by Rousseau et al. [39].

Figure 2d shows the stability of the ISE. In this water layer test, 100 mM NaNO_3_ was used as the primary solution, and 100 mM NaCl was the interfering solution. First, the ISE was conditioned in NaNO_3_ until it was stable. The final hour of stable output in NaNO_3_ is shown, followed by two hours in the interfering solution, and returning to NaNO_3_ for 24 h. The potential shows some drift during both the NaCl step and the NaNO_3_ return, which could indicate the presence of a water layer on the electrode’s surface, which is not unexpected for this type of coated-wire electrode. However, the electrode’s stability is on par with values reported in the literature, which involved specific modifications for stability. The difference between the potential immediately before and the potential immediately after the NaCl step is 15 mV, the same as found by Chen for electrodes using gold nanoparticles and Polypyrrole (PPy) to improve stability [31]. The drift over time for our electrodes is 0.7 mV/h, which is comparable to the 0.8 mV/h and 0.9 mV/h for screen-printed electrodes reported by Jiang and Fan, respectively [24,26].

### 3.2. Reference Electrode Development

Reference electrodes act as electrochemical ground; therefore, their potential must remain unchanged in varying ionic environments. The precise composition of the printed RE will impact $E_{0}$ in the Nernst equation. However, because $E_{0}$ is constant, the offset is easily accounted for in calibration.

**Figure 3:**
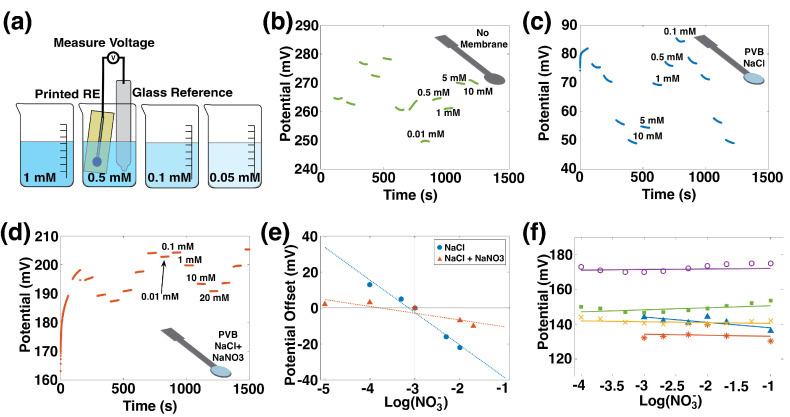
(**a**) Measuring a printed reference electrode against a commercial reference electrode in NaNO_3_ solutions of varying concentrations. Potential over time in changing concentrations of nitrate of a printed Ag/AgCl reference electrode with (**b**) no added membrane, (**c**) PVB membrane with NaCl added, and (**d**) PVB membrane with NaCl and NaNO_3_ added. Measurements in (**b**–**d**) were done against a commercial Ag/AgCl reference electrode. (**e**) Sensitivity of printed reference electrodes with NaCl in PVB membrane (blue), and NaNO_3_ and NaCl in PVB membrane (red). The absolute value of the voltage measured at 1 mM NaNO_3_ has been set to 0 mV to facilitate comparison of slopes. (**f**) Sensitivity of five printed reference electrodes to NO_3_ is 2.96 ± 1.9 mV/dec.

The performance of printed REs was determined by measuring them versus a commercial Ag/AgCl double junction RE, as in Zamarayeva [40] and illustrated in Figure 3a. First, pristine printed Ag/AgCl electrodes were measured, and the resulting data are shown in Figure 3b. The output voltage is unstable since these printed REs lack a source of chloride ions, which are needed for the Ag/AgCl reversible reaction:

(2) $$\mathrm{AgCl}\;+\;e^{-}⇌\mathrm{Ag}\;+\;{\mathrm{Cl}}^{-}$$

The surface area and composition of the printed RE were modified by adding a CNT layer and a PVB-NaCl membrane was added to provide a source of chloride. The characterization is shown in Figure 3c. These electrodes used the formulation developed in Zamarayeva [40] for use in chloride-rich environments. REs with an NaCl membrane show a −18 mV/dec sensitivity to nitrate, which is unacceptably high.

The optimized RE composition was achieved with the addition of NaNO_3_ to the PVB-NaCl membrane. Cattrall and Zamarayeva et al. [40,41] have shown that including the ion of interest in the membrane of an RE reduces its sensitivity to that ion. To reduce sensitivity to nitrate, NaNO_3_ was added to the membrane; sensitivity data for this electrode are shown in Figure 3d. This formulation has a sensitivity of −3 mV/dec, which is a marked improvement over the NaCl membrane alone.

The effect of adding the ion of interest to the reference electrode membrane is highlighted in Figure 3e, where the NaCl membrane and NaCl+NaNO_3_ membranes are directly compared. In this figure, potentials are normalized by subtracting the average potential in 1 mM nitrate from the average potential at each concentration, and the potential offsets are plotted versus nitrate concentration. The RE whose membrane includes NaCl + NaNO_3_ has a flatter slope, which reflects its insensitivity to nitrate concentration.

Repeatability across different reference electrodes is shown in Figure 3f, where voltage versus concentration for five printed REs with the NaCl + NaNO_3_ + PVB membranes is displayed. All the printed REs showed a stable potential response over three orders of magnitude change in the nitrate concentration.

### 3.3. Interference

Soil is a complex environment containing a host of ions other than ${\mathrm{NO}}_{3}^{-}$. Ideally, ${\mathrm{NO}}_{3}^{-}$ ISEs should be insensitive to all ions other than ${\mathrm{NO}}_{3}^{-}$, and REs should be stable regardless of the concentration of any ion. Selectivity studies quantify the degree to which these behaviors are true and identify elements which could cause errors in the measurements.

The Nicolsky–Eisenman equation describes the potential, E, generated by a potentiometric sensor in the presence of interfering species [42].

(3) $$E=E_{0}+2.3026\frac{RT}{zF}log_{10}\left(a_{A}+\sum_{B}K_{A,B}^{POT}{\left(a_{B}\right)}^{\frac{z_{A}}{z_{B}}}\right)$$

It assumes Nernstian behavior for all ions, and interfering species’ responses are weighted by their respective Nicolsky–Eisenman coefficient, $K_{A,B}^{POT}$, where *A* is the primary ion (${\mathrm{NO}}_{3}^{-}$, in this case) and *B* is the interfering species. $K_{A,B}^{POT}$ should be less than 1, and the nearer to zero, the less sensitive the ISE is to that interfering species.

Based on a soil chemistry report from A & L Western Agricultural Laboratories, eight possibly interfering species were chosen: sulphate (${\mathrm{SO}}_{4}^{2-}$), chloride (Cl^−^), phosphate (${\mathrm{PO}}_{4}^{3-}$), nitrite (${\mathrm{NO}}_{2}^{-}$), ammonium (${\mathrm{NH}}_{4}^{+}$), calcium (${\mathrm{Ca}}^{2+}$), potassium ($K^{+}$), and magnesium (${\mathrm{Mg}}^{2+}$). Higher concentrations of ${\mathrm{SO}}_{4}^{2-}$ and ${\mathrm{Cl}}^{-}$ were also tested because they rank above ${\mathrm{NO}}_{3}^{-}$ in the Hoffmeister series, so are of particular concern as interfering species. The concentrations of these chemicals and the salt used as the source of the ions are listed in Table 2.

The two-solution method, which is a mixed solution method, was used to determine the $K_{A,B}^{POT}$ values of the ISEs for the ions listed above [42]. Printed ISEs were paired with a commercial Ag/AgCl reference electrode. Then, measurements were recorded first in 1mM NaNO_3_ and then interfering salt and 1 mM NaNO_3_. The difference in potential, Δ*E*, was used in Equation (4) to calculate $K_{A,B}^{POT}$.

(4) $$K_{A,B}^{POT}=a_{A}\left(e^{\Delta Ez_{A}F/\left(RT\right)}-1\right)/{\left(a_{B}\right)}^{z_{A}/z_{B}}$$

We also measured the printed REs’ response to interfering ions by measuring them against commercial glass electrodes, first in 1mM NaNO_3_ and then interfering salt and 1 mM NaNO_3_. Because REs should not have Nernstian responses to ions, Equation (3) is not a good model for RE behavior. Instead, simple Δ*E* values are reported in Table 2.

As shown in Table 2, the $K_{A,B}^{POT}$ values for the ISEs and Δ*E* values for REs are quite small for most ions except ${\mathrm{Ca}}^{2+}$ at concentrations that are expected in soil. ${\mathrm{Ca}}^{2+}$, however, has a significant impact on both the ISE and the RE, indicating that in soils with high concentrations of calcium, the sensor might be unreliable, or at least require site-specific calibration.

In addition to being insensitive to interfering ions, their presence should not lower the sensitivity of the ISEs to ${\mathrm{NO}}_{3}^{-}$. The sensitivity of four sensors was measured between 0.1 and 100 mM concentrations of KNO_3_ and NH_4_NO_3_ fertilizers, and 0.05 to 50 mM Ca(NO_3_)_2_. The sensitivities in KNO_3_and NH_4_NO_3_ fertilizers were −52.6 ± 5 mV/dec and −51.1 ± 4 mV/dec, respectively, but −29.3 ± 10.6 mV/dec in Ca(NO_3_)_2_. The impact of ${\mathrm{Ca}}^{2+}$ on sensor behavior is important and deserves further study because ${\mathrm{Ca}}^{2+}$ can be present at high concentrations in soil, and is used in fertilizers as well.

### 3.4. Fully Printed Sensors

**Figure 4:**
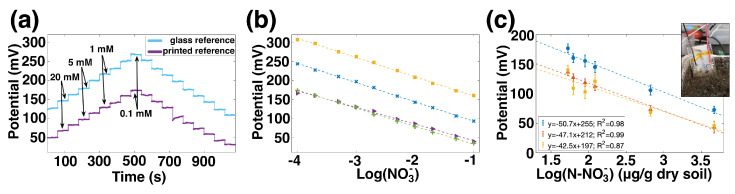
(**a**) Potential over time for an ISE measured against a commercial glass reference electrode (light blue) and against a printed reference electrode (dark purple). The change in reference electrode changed the $E_{0}$ of the pair by 87 mV. (**b**) Sensitivity curves for printed sensors from two different batches. The average sensitivity for these four sensors is 48.0 ± 3.3 mV/dec. (**c**) Potential versus nitrate concentration for three sensors in a high-organic-matter soil.

Pairing the printed ISE with a printed RE results in a fully printed sensor which realizes the benefits of printing: low cost, high-throughput manufacturing, no glass or liquid components, and production in form factors that are suitable for use in field deployments. Figure 4a shows the potential over time for a printed ISE measured against a commercial reference in light blue, and the same ISE paired with a printed reference in dark purple. The $E_{0}$ value has changed, which is expected because the interfaces present in a printed RE are different from those of a commercial RE. For this sample, the fully printed sensor’s potential is approximately 87 mV below the printed ISE–commercial RE pair. Both versions have high sensitivity over the 0.1 mM to 100 mM range, response times less than 10 s, and hysteresis less than 5%.

The sensitivity of four such printed sensors, from two batches, is shown in Figure 4b. The sensitivity of these ISEs when measured against glass REs was −54.3 ± 2.6 mV/dec, which is near Nernstian and comparable to other nitrate ISEs in Table 1. When the glass references were replaced with printed references, the sensitivity decreased slightly to 48.0 ± 3.3 mV/dec. This decrease in sensitivity can be attributed to the slight sensitivity of the printed reference electrodes to nitrate. Again, $E_{0}$ variation is considerable, particularly from batch to batch. This is expected given the batch-to-batch variability of the ISEs and the sample-to-sample variation of printed REs.

Fully printed nitrate sensors were measured in high-organic-matter soil from a field site in California. Six small pots of soil were prepared; each was saturated with a different concentration of KNO_3_ solution. The printed sensors were inserted into each pot in turn, and the potential recorded. Plant-available NO_3_ concentration—including background NO_3_ already present in the soil prior to watering—was measured using standard techniques of KCl extraction and colorimetric analysis of the extracted liquid described in the Materials and Methods section.

Figure 4c shows the relationship between the sensors’ potential and the log of the concentration of nitrate, which was linear with R^2^ values of 0.98, 0.99, and 0.87. The average sensitivity was −47 mV/dec, which was similar to their sensitivity in aqueous solution. This is an important result because it shows that the sensitivity of the NO3 ISEs in direct soil application—rather than slurries or percolates—can be as good as their sensitivity in solution. The $E_{0}$ variation means that each sensor would need individual calibration to provide absolute accurate measurements, rather than relative changes; this is a challenge common to ISEs, including commercial nitrate probes. These results are promising for the future application of printed ISEs in soil media.

## 4. Conclusions

We designed and fabricated fully printed potentiometric nitrate sensors comprising a printed nitrate ISE and printed RE. The printed nitrate ISEs showed a near-Nernstian sensitivity of −54.1 ± 2.1 mV/dec when paired with a glass RE. A printed RE with low sensitivity to nitrate was developed using a membrane composed of PVB, NaCl, and NaNO_3_. Fully printed nitrate sensors demonstrated sensitivity of −48.0 ± 3.3 mV/dec in solution and −47 mV/dec in soil. Printed sensors were not significantly impacted by sulphate, chloride, phosphate, nitrite, ammonium, potassium, and magnesium at concentrations expected in soil, but calcium did interfere with sensor behavior.

The fabrication methods used here are scalable and relatively low-cost when compared to conventional electronics. Because the sensors are passive, they would require little power to be read, which is advantageous when integrating into wireless sensor nodes. As a result, they could be widely distributed throughout a landscape to map the movement of nitrate through the watershed, inform efficient application of fertilizer, or alert residents to elevated nitrate levels in drinking water.

## Figures and Tables

**Table 1:** Comparison of sensitivity, selectivity, and reference electrodes for nitrate-selective potentiometric sensors.

Fabrication Technique	ISE Materials	Sensitivity (mV/dec)	Selectivity Ions	Reference Electrode Materials	Reference Electrode Characterization	Application	Reference
Screen print,	AgCl,	−54	H_2_${\mathrm{PO}}_{4}^{-}$, ${\mathrm{SO}}_{4}^{2-}$,	Commercial		Soil extraction	[23]
Drop cast	gel		${\mathrm{NO}}_{2}^{-}$, ${\mathrm{CO}}_{2}^{-}$				
Screen print	PTFE membrane	−57.2	H_2_${\mathrm{PO}}_{4}^{-}$, ${\mathrm{SO}}_{4}^{2-}$,	Not		Wastewater	[24]
			Cl^−^	specified			
Drawing,	Pencil	−49.4	${\mathrm{SO}}_{4}^{2-}$, Cl^−^,	Commercial		Soil extraction	[25]
Drop cast	Graphite		${\mathrm{NO}}_{2}^{-}$, ${\mathrm{OH}}^{-}$				
Stencil print	Silver	−57	H_2_${\mathrm{PO}}_{4}^{-}$, ${\mathrm{SO}}_{4}^{2-}$,	Ag/AgCl		Soil slurry,	[26]
			Cl^−^	Paste		Pulses in soil	
Laser,	LIG	−54.8		Ag/AgCl		Soil slurry,	[27]
Drop cast				paint		Pulses in soil	
Evaporation,	Gold/	−64	${\mathrm{PO}}_{4}^{3-}$, ${\mathrm{SO}}_{4}^{2-}$, ${\mathrm{Cl}}^{-}$,	Screen printed	versus Cl^−^	Soil Slurry,	[28]
Dispenser robot	POT-MOS_2_		${\mathrm{NO}}_{2}^{-}$, ${\mathrm{HCO}}_{3}^{-}$	Ag/AgCl with Nafion		Pulses in soil	
	Glassy carbon	−57.9	${\mathrm{SO}}_{4}^{2-}$, ${\mathrm{Cl}}^{-}$	Commercial		Drinking water	[29]
	Graphene						
Electrodeposition	Au-NP and PPy	−50.4	${\mathrm{PO}}_{4}^{3-}$, ${\mathrm{SO}}_{4}^{2-}$,	Commercial			[30]
	on glassy carbon		Cl^−^, Br^−^				
Electrodeposition	Au-NP, PPy	−50	$H_{2}$${\mathrm{PO}}_{4}^{-}$, ${\mathrm{SO}}_{4}^{2-}$,	Commercial		Soil Percolate	[31]
	and graphene oxide		CH_3_COO, HCO_3_				
Drop cast	Graphene/TTF	−59.1		Commercial			[32]
	on glassy carbon						
	CNT/ionic	−52.3 to	$H_{2}$${\mathrm{PO}}_{4}^{-}$, ${\mathrm{SO}}_{4}^{2-}$,	Commercial			[33]
	liquid on	−57.1	Cl^−^, ${\mathrm{NO}}_{2}^{-}$, ${\mathrm{CO}}_{3}^{-}$				
	glassy carbon		${\mathrm{CH}}_{3}$COO, F^−^, Br^−^				
Electrodeposition	PPy on	−54.1		Commercial		Pulses in	[34]
	wire					soil	
Inkjet and	Gold	−54.1	${\mathrm{PO}}_{4}^{3-}$, ${\mathrm{SO}}_{4}^{2-}$, Cl^−^,	Screen printed	versus ${\mathrm{NO}}_{3}^{-}$,	Field soil	This Work
screen print			${\mathrm{NO}}_{2}^{-}$, ${\mathrm{NH}}_{4}^{+}$, ${\mathrm{Ca}}^{2+}$	Ag/AgCl with CNT	other ion	sensitivity	
			$K^{+}$, ${\mathrm{Mg}}^{2+}$	and NaCl/NaNO_3_	interference		

**Table 2:** Nickolsy–Eisenman coefficients for ions found in soil.

Chemical (ppm)	Concentration	Concentration and Salt Used	$K_{A,B}^{\mathrm{POT}}$ for ISE	Δ*E* for RE (mV)
Sulphate	20 ppm	0.2 mM Na_2_SO_4_	−0.087	−0.67
Sulphate	96 ppm	1 mM Na_2_SO_4_	−0.019	−4.33
Chloride	35.5 ppm	1 mM NaCl	0.064	0.33
Nitrite	30 ppm	0.65 mM NaNONa_2_	0.086	−0.67
Ammonium	10 ppm	0.55 mM NH_4_Cl	0.012	−0.67
Potassium	600 ppm	15.3 mM KCl	0.317	−2.33
Magnesium	400 ppm	16.5 mM MgCl	0.004	3.67
Phosphate	20 ppm	0.2 mM Na_3_PO_4_	0.074	2.00
Chloride	5300 ppm	150 mM NaCl	0.002	2.67
Calcium	3000 ppm	75 mM CaCl_2_	1.377	12.67

## Data Availability

Not applicable.
